# The impact of structural factors on diagnostic delay in diffuse large B‐cell lymphoma

**DOI:** 10.1002/cam4.2009

**Published:** 2019-03-18

**Authors:** Joanna C. Zurko, Raymond C. Wade, Amitkumar Mehta

**Affiliations:** ^1^ Department of Medicine University of Alabama at Birmingham Birmingham Alabama; ^2^ Division of Hematology and Oncology University of Alabama at Birmingham Birmingham Alabama

**Keywords:** delayed diagnosis, diffuse, humans, large B‐Cell, lymphoma, medically uninsured, non‐hodgkin, retrospective studies

## Abstract

**Background:**

Reducing diagnostic delays in cancer has been a major interest worldwide; however, the literature on diagnostic delays in lymphoma remains scarce. Diffuse large B‐cell lymphoma (DLBCL) is the most common non‐Hodgkin's lymphoma. We aimed to determine whether certain structural factors predicted diagnostic delays in DLBCL and whether diagnostic delays impacted overall survival (OS).

**Methods:**

Data were extracted via a retrospective cohort design from a single academic tertiary care referral center. A total of 104 patients were included. Time from first symptoms to diagnosis of <3 months was defined as “early diagnosis” and ≥3 months as “delayed diagnosis”. Analysis was performed with student's *t*‐test, chi‐square testing, binomial logistic regression, and Kaplan‐Meier log‐rank testing.

**Results:**

“Delayed diagnosis” was more likely with lower stage, lower international prognostic index (IPI), and further distance from referral center (OR 0.66, CI 0.46‐0.95; OR 0.69, CI 0.51‐0.94; OR 1.008, CI 1.001‐1.015). Patients of “other” ethnicity and without medical insurance were more likely to have significant diagnostic delays and worse overall survival (*P* = 0.002 and *P* = 0.007, respectively). Diagnostic delays of ≥3 months did not predict worse OS. However, delays of >6 months did predict worse OS.

**Conclusion:**

Our data suggest that excessive diagnostic delays of more than 6 months, ethnic minority status, and uninsured status in DLBCL may lead to worse outcomes. Efforts should be undertaken to reduce excessive diagnostic delays. More investigation needs to be done on the impacts of diagnostic delays in both DLBCL and other aggressive lymphomas.

## 1 INTRODUCTION

There has been considerable interest over the past several decades in reducing diagnostic delays in patients with cancer in an attempt to reduce cancer‐related mortality.^1^, ^2^, ^3^ While there is a relative abundance of data on diagnostic delays in solid tumor malignancies, there is a paucity of data on diagnostic delays in hematologic malignancies, especially lymphoma.^4^, ^5^, ^6^, ^7^, ^8^ The different subtypes of lymphoma have significantly different presentations, treatments and prognosis; therefore, focusing on a specific subtype of lymphoma is ideal when assessing the prevalence and impacts of diagnostic delays. Non‐Hodgkin's lymphoma (NHL) is the seventh most common cancer in the United States and diffuse large B‐cell lymphoma (DLBCL) is the most common NHL accounting for a third of all cases.^9^, ^10^ Patients most commonly present with a rapidly enlarging mass and about a third present with B‐symptoms.^11^ Five‐year overall survival is 60.5% and prognosis depends primarily on stage and international prognostic index (IPI), but is also affected by other factors such as bulky disease and bone marrow involvement.^12^ Our main objective was to determine whether insurance status and distance from referral center predicted delayed diagnosis in DLBCL and to determine whether diagnostic delays had an impact on overall survival.

## 2 METHODS

Patients seen at the University of Alabama at Birmingham (UAB) Hospital and Clinics between 2009 and 2013 with a new pathologic diagnosis of DLBCL were identified (n = 180). Data were collected through retrospective chart review. Patients were excluded if there was inadequate follow‐up available in the chart (for example, if a patient was diagnosed at our institution but then was treated and followed up for their DLBCL at an outside institution) or if patients had a diagnosis of primary central nervous system (CNS) lymphoma (n = 76). A time from first symptoms to pathologic diagnosis of <3 months was defined as “early diagnosis” whereas time from symptoms to diagnosis ≥3 months was defined as “delayed diagnosis”. We defined time of diagnosis as time of confirmed pathologic diagnosis. Categorical and continuous variables were compared using student's *t*‐test and chi‐square testing. Binomial logistic regression was performed to assess for predictors of delayed diagnosis and overall survival. Variables assessed included age, international prognostic index (IPI), stage, ethnicity, distance from tertiary care referral center, Charlson Comorbidity Index (CCI), having an established primary care physician (PCP), initially presenting to a primary care physician's office, having medical insurance, presence of bulky disease, B‐symptoms, bone marrow involvement, disease status at last follow‐up, 3‐year survival, and overall survival. Ethnicity was recorded as white, black or other in data collection. Overall survival curves were constructed using Kaplan‐Meier method log‐rank (Mantel‐Cox) testing. Statistical analysis was done using SPSS Statistics 25. All *P*‐values were two sided and a *P*‐value <0.05 was considered significant.

## 3 RESULTS

### 3.1 Baseline characteristics

A total of 104 patients were included in the analysis. Time to diagnosis and overall survival based on clinical characteristics are shown in Table 1. In our cohort, median age was 63.5 years, 46.2% of patients were female, 76.0% of patients identified as “white” ethnicity, 19.2% as “black” and 4.8% as “other”. Patients of “other” ethnicity included patients of Chinese, Egyptian, Indian, and Mexican descent only one of which required interpreter services. It was clear based on chart review that at least 4 of the 5 patients of “other” ethnicity were originally born outside of the United States. We were unable to determine this based on chart review for one of the patients included in the analysis. 38.5% of patients resided in a rural county. 12.5% of patients had no medical insurance. The mean distance traveled by patients to the tertiary care referral center where they received their cancer care was 64.5 miles. On average patients took 4.0 months from first symptoms to diagnosis, 3.4 months from their first symptoms to being evaluated by a subspecialist at a tertiary care referral center and 19.3 days to be diagnosed after seeing a subspecialist. Mean time of follow‐up after diagnosis was 3.3 years. 66.4% of patients were in complete remission at last follow‐up. Three‐year overall survival was 67.9% and overall survival at last follow‐up was 66.4%.

**Table 1:** Time from first symptoms to diagnosis (months) and overall survival (%) among all patients stratified by clinical characteristics

	Number of patients	Time from symptoms to diagnosis (months), mean (SD)	*P*‐value	Overall survival, %	*P*‐value
Age (years)					
>60	62	3.7 (4.4)	0.56	64.5	0.20
≤60	42	4.4 (7.5)		76.2	
Gender					
Female	48	4.1 (4.1)	0.86	70.8	0.74
Male	56	3.9 (7.0)		67.9	
Ethnicity					
White	79	3.6 (3.6)	0.18^a^	69.6	0.36^a^
Black	20	2.4 (2.0)		80.0	
Other	5	16.8 (19.5)	<0.001^b^	20.0	*0.015* b
County of residence					
Rural	40	3.6 (3.1)	0.56	67.5	0.76
Urban	64	4.3 (7.0)		70.3	
Charlson comorbidity index					
0‐2	57	4.4 (7.0)	0.39	71.9	0.51
3‐8	47	3.4 (3.9)		66.0	
Distance traveled to tertiary care referral center (miles)					
≤50 miles	47	3.9 (7.5)	0.90	70.2	0.95
>50 miles	56	4.1 (4.0)		69.6	
Have an established primary care physician					
No	27	5.8 (9.8)	0.064	59.3	0.19
Yes	77	3.4 (3.3)		72.7	
Have medical insurance					
No	13	7.7 (13.8)	0.013	46.2	0.055
Yes	91	3.5 (3.3)		72.5	
Initial presentation at primary care physician office					
No	54	4.3 (7.2)	0.54	64.8	0.31
Yes	50	3.6 (3.7)		74.0	
International prognostic index (IPI)					
IPI low (0‐1)	30	5.5 (8.5)	0.12^c^	90.0	<0.001^d^
IPI low intermediate (2)	27	3.2 (2.2)		81.5	
IPI high intermediate (3)	24	3.2 (5.0)		70.8	
IPI high (4‐5)	19	4.0 (5.1)		21.1	
Stage of disease					
Limited stage (1‐2)	36	5.0 (7.8)	0.20	83.3	0.024
Advanced stage (3‐4)	68	3.5 (4.3)		61.8	
Bone marrow involvement					
No	78	4.0 (6.0)	0.76	75.64	0.81
Yes	7	3.3 (2.4)		71.43	
Bulky disease					
No	77	4.3 (6.6)	0.34	72.7	0.19
Yes	27	3.1 (2.5)		59.3	
B symptoms					
No	72	3.9 (6.3)	0.83	76.4	0.02
Yes	32	4.2 (4.6)		53.1	

a Comparing “white” vs “black” ethnicity.

b Comparing “white and black” vs “other” ethnicity.

c Comparing international prognostic index of 0‐1 vs 2‐5.

d Comparing international prognostic index of 0‐3 vs 4‐5.

### 3.2 Characteristics of patients with early versus delayed diagnosis

A total of 55 patients were categorized as having “early diagnosis” (time from symptoms to diagnosis <3 months) and 49 patients had “delayed diagnosis” (≥3 months). Characteristics of patients with early vs late diagnosis are summarized in Table 2. There was no statistically significant difference in age, gender, rural county residence, Charlson Comorbidity Index (CCI), insurance status, having an established PCP or initial presentation to a PCP office between groups. There was also no difference in rates of bulky disease, B symptoms, bone marrow involvement, overall survival, progression at last follow‐up or complete remission at last follow‐up early and delayed diagnosis groups. Overall survival was 69.1% and 69.4% in patients with early vs delayed diagnosis (*P* = 0.97), respectively. Patients who had “early diagnosis” had a higher IPI (2.5 vs 1.9, *P* = 0.014) and a higher stage (3.2 vs 2.7, *P* = 0.024) than those with “delayed diagnosis”. Patients with “early diagnosis” also had higher rates of receiving their initial chemotherapy as an inpatient (35.2% vs 6.7%, *P* = 0.0007). Patients who had “delayed diagnosis” lived farther from the tertiary care referral center (81.0 vs 49.5 miles, *P* = 0.015) and had a higher proportion of patients who identified as “other” ethnicity (10.2% vs 0.0%, *P* = 0.016).

**Table 2:** Characteristics of patients with early vs late diagnosis (<3 months vs ≥3 months from first symptoms to pathologic diagnosis)

	Early diagnosis (<3 months) n = 55	Delayed diagnosis (≥3 months) n = 49	*P*‐value
Age (years), mean (median)	61.1 (64.0)	60.4 (63.0)	0.81
Female, n (%)	22 (40.0%)	26 (53.1%)	0.18
White ethnicity, n (%)	41 (74.5%)	38 (77.6%)	0.72
Black ethnicity, n (%)	14 (25.5%)	6 (12.2%)	0.090
Other ethnicity, n (%)	0 (0.0%)	5 (10.2%)	0.016
Distance from tertiary care referral center (miles), mean (SD)	49.5 (47.1)	81.0 (79.8)	0.015
Charlson Comorbidity Index (CCI), mean (SD)	2.5 (1.7)	2.6 (1.8)	0.73
Had an established PCP at diagnosis, n (%)	42 (76.4%)	35 (71.4%)	0.57
Had medical insurance at diagnosis, n (%)	47 (85.5%)	44 (89.8%)	0.51
IPI, mean (SD)	2.5 (1.4)	1.9 (1.3)	0.014
Stage, mean (SD)	3.2 (1.0)	2.7 (1.1)	0.024
Initial presentation at PCP office, n (%)	25 (45.5%)	25 (51.0%)	0.57
Initial chemotherapy received inpatient, n (%)	19^a^ (35.2%)	3^b^ (6.7%)	<0.001
Chemotherapy never given, n (%)	1 (1.8%)	4 (8.2%)	0.13
Bone marrow involvement, n (%)	4^c^ (9.1%)	3^d^ (7.3%)	0.77
Bulky disease, n (%)	15 (27.3%)	12 (24.5%)	0.75
B symptoms, n (%)	14 (25.5%)	18 (36.7%)	0.22
Residing in rural county, n (%)	34 (61.8%)	30 (61.2%)	0.95
Complete remission at last follow‐up, n (%)	36 (65.5%)	33 (67.3%)	0.84
Progression at last follow‐up, %	7 (12.7%)	5 (10.2%)	0.69
3‐year overall survival	31^e^ (68.9%)	26^f^ (66.7%)	0.83
Overall survival last follow‐up	38 (69.1%)	34 (69.4%)	0.97

a Out of 54 patients.

b Out of 45 patients.

c Out of 44 patients.

d Out of 41 patients.

e Out of 45 patients.

f Out of 39 patients.

### 3.3 Predictors of delayed diagnosis

On binomial logistic regression, stage, IPI, and distance from tertiary care center significantly predicted delayed diagnosis (Table 3). A higher stage and higher IPI score predicted a decreased likelihood of delayed diagnosis (OR 0.66, 95% CI 0.46‐0.95, *P* = 0.03; OR 0.69, 95% CI 0.51‐0.94, *P* = 0.02, respectively). An increased distance (in miles) from the tertiary care referral center predicted an increased likelihood of delayed diagnosis (OR 1.008, 95% CI 1.001‐1.015, *P* = 0.023). Delayed diagnosis predicted a decreased likelihood of receiving first chemotherapy inpatient (OR 0.13, CI 0.04‐0.48, *P* = 0.002). Age, rural county residence, insurance status, B symptoms, bulky disease, having an established primary care physician (PCP) and presenting initially to a PCP office did not significantly predict early vs delayed diagnosis.

**Table 3:** Predictors of delayed diagnosis (time from first symptoms to diagnosis ≥3 months) using binomial logistic regression

	OR (95% CI)	*P*‐value
Age (years)	1.00 (0.97‐1.02)	0.81
Gender, female	1.70 (0.78‐3.69)	0.18
Have an established primary care physician at diagnosis	0.77 (0.32‐1.86)	0.57
Rural county residence	1.03 (0.47‐2.26)	0.95
Charlson Comorbidity Index	1.04 (0.83‐1.30)	0.73
Distance from tertiary care referral center (miles)	1.01 (1.001‐1.02)	0.023
Had health insurance at diagnosis	1.50 (0.46‐4.93)	0.51
Initially present to primary care physician office	1.25 (0.58‐2.70)	0.57
International prognostic index (IPI)	0.69 (0.51‐0.94)	0.019
Stage	0.66 (0.46‐0.95)	0.027
B symptoms	1.70 (0.73‐3.94)	0.22
Bulky disease	0.87 (0.36‐2.09)	0.75
First chemotherapy inpatient	0.13 (0.036‐0.48)	0.002
Complete remission at last follow‐up	1.09 (0.48‐2.46)	0.84
Progression at last follow‐up	0.78 (0.23‐2.64)	0.69

### 3.4 Characteristics of patients that identified as “other” ethnicity and uninsured patients

Patients that identified as “other” ethnicity had a significantly longer time to diagnosis and had worse overall survival on average than patients who identified as white or black (Table 1). Patients of “other” ethnicity took a mean of 16.8 months (median of 5.0 months) from symptoms to diagnosis compared to 3.6 months for “white” patients and 2.4 months for black patients (*P* < 0.0001). Overall survival (OS) was 20.0% for patients of “other” ethnicity vs 69.6% for “white” and 80.0% for “black” patients (*P* = 0.015). Patients of “other” ethnicity had higher rates of having no medical insurance (40.0% for other vs 11.4% for white and 10.0% for black patients, *P* = 0.04). IPI scores were higher in patients of “other” ethnicity but this did not reach statistical significance (3.2 for “other” vs 2.2 for “white” versus 2.1 for “black”, respectively, *P* = 0.10). There was no statistically significant difference in age, CCI, stage, or distance from tertiary care referral center between groups.

Patients without medical insurance at time of diagnosis also took significantly longer from time of first symptoms to pathologic diagnosis and had worse 3‐year overall survival than insured patients in our cohort. Uninsured patients took a mean of 7.7 months from symptoms to diagnosis compared to a mean of 3.5 months in patients with health insurance (*P* = 0.013). Three‐year overall survival was 33.3% in patients without medical insurance compared with 72.0% in patients with insurance (*P* = 0.020). Differences in overall survival at last follow‐up between those without and with medical insurance neared but did not reach statistical significance (46.15% vs 72.53%, respectively, *P* = 0.055). Differences in prevalence of “other” ethnicity between those without and with medical insurance also neared but did not reach statistical significance (15.4% vs 3.3%, respectively, p = 0.057). There was no significant difference in age, CCI, stage, IPI or distance from tertiary care referral center between groups.

### 3.5 Survival analysis and predictors of overall survival

On Kaplan‐Meier survival analysis log‐rank (Mantel‐Cox) testing, time from symptoms to diagnosis <3 months versus ≥3 months and ≤5 months vs >5 months had no significant impact on survival (*P* = 0.88 and *P* = 0.57, respectively). Nonetheless, patients that took >6 months from symptoms to diagnosis compared to ≤6 months had significantly worse overall survival with an OS of 44% vs 72%, respectively (*P* = 0.009 on Kaplan‐Meier log‐rank testing). Patients who had B symptoms at diagnosis, identified as “other” ethnicity and did not have medical insurance had worse overall survival on Kaplan‐Meier survival analysis (*P* = 0.044, *P* = 0.002 and *P* = 0.007, respectively). On binomial logistic regression, higher stage, IPI, and CCI all predicted death at last follow‐up (OR 1.96 [1.24‐3.12], *P* = 0.004; OR 2.89 [1.79‐4.67], *P* < 0.001; OR 1.51 [1.15‐1.97], *P* = 0.003, respectively).

## 4 DISCUSSION

In our analysis, patients with a lower stage and International Prognostic Index were more likely to have a delayed diagnosis. This could be related to patients with more aggressive disease presenting to the health system and being referred earlier due to increased severity of symptoms. Residing further from a tertiary care referral center also predicted delayed referral which has not previously been assessed in patients with lymphoma. Relatively brief diagnostic delays (<3 months vs ≥3 months) were not associated with worse overall survival on our analysis. Moreover, in our cohort, a time to diagnosis of >6 months was associated with significantly worse survival. This suggests there may be a threshold at which excessive diagnostic delays in DLBCL may lead to worse outcomes. While it is well known that later stage at diagnosis is associated with worse outcomes in cancer, the literature is mixed in concluding whether a reduction in diagnostic delays will lead to improved outcomes in all cancer types. For example, in women with breast cancer, diagnostic delays of ≥3 months were shown to negatively impact overall survival.^13^ Similarly, diagnostic delays have been shown to negatively impact outcomes in head and neck cancer.^14^ Conversely, diagnostic delays in symptomatic colon cancer, gynecologic malignancies, and Hodgkin's lymphoma have not been shown to negatively impact survival.^8^, ^15^, ^16^, ^17^

Both uninsured patients and those who identified as “other” ethnicity had worse survival as well as longer time to diagnosis in our cohort. Our findings are consistent with prior studies in the literature showing that ethnic minorities and patients without medical insurance who are diagnosed with cancer suffer worse overall outcomes.^18^, ^19^ Being uninsured at the time of diagnosis with non‐Hodgkin's lymphoma has been shown to significantly increase the risk of advanced stage at diagnosis which in turn is associated with worse overall survival.^18^ Uninsured patients face significant barriers to access to care. These patients often seek care in emergency departments and are often referred for follow‐up appointments they cannot make or afford. A United States survey of physicians noted that 40.3% of physicians did not accept “no charge” or charity patients in their clinic.^20^ Another study showed that when trying to find follow‐up after being seen in the ER for a serious medical condition, only a quarter of uninsured patients were given appointments.^21^ Patients that made up the “other” ethnicity cohort in our study included patients primarily of Hispanic and Asian descent. These groups are both ethnic minorities in our state making up 4.2% and 1.4% of the population, respectively.^22^ There was significant overlap in the patients that were uninsured and that identified as “other” ethnicity as patients of “other” ethnicity were significantly more likely to have no health insurance. This is consistent with other studies showing that ethnic minorities were to more likely to be uninsured than white patients with cancer.^18^ In our cohort patients of “other” ethnicity trended toward even worse outcomes than patients who were uninsured, with longer time to diagnosis and worse overall survival. Other studies have shown that ethnicity remains a significant predictor of advanced cancer stage at diagnosis even after controlling for insurance status.^18^ It is probable that language and cultural factors affect how ethnic minorities seek care in our region and also affect patient‐physician communication. In our cohort several of our patients were immigrants and non‐Native English speakers which could have contributed to some of these worse outcomes.

Our study defined diagnostic delays based on time from first symptoms to pathologic diagnosis. Prior studies assessing time to diagnosis in lymphoma have looked primarily at the time from first health care contact to diagnosis (assessing system‐related delays) when defining what constitutes a diagnostic delay.^4^, ^5^, ^7^ This does not account for the impacts of time from first symptoms to first health care contact, which is most commonly known as the “patient‐related delay” in the literature.^23^ Prior policies in places like the United Kingdom (UK) have primarily focused on reducing referral delays in patients with suspected cancer and have not focused on patient‐related delays.^24^ Nonetheless, the primary driver of diagnostic delays in lymphoma has been shown in several studies to be the patient‐related delay and not system‐related delays.^4^, ^5^, ^6^, ^7^ If diagnostic delays do in fact lead to worse outcomes in lymphoma, focusing only on improving issues such as referral delays neglects a key driver of these worse outcomes.

There were several limitations to our study. First, our study was relatively small with a total of 104 patients. Our data were subject to potential recall bias since the time of first symptoms was obtained through what was recorded in patient records. The absence of a single definition of what constitutes diagnostic delay for DLBCL in the literature made defining diagnostic delay in our study difficult.

In summary, our data suggest that excessive diagnostic delays in diffuse large B‐cell lymphoma may lead to worse outcomes for patients. More investigation needs to be done on the impacts of diagnostic delays in both DLBCL and other aggressive lymphomas.

## CONFLICT OF INTEREST

There are no conflicts of interest to disclose from any of the authors.
